# BenchStab: a tool for automated querying of web-based stability predictors

**DOI:** 10.1093/bioinformatics/btae553

**Published:** 2024-09-11

**Authors:** Jan Velecký, Matej Berezný, Milos Musil, Jiri Damborsky, David Bednar, Stanislav Mazurenko

**Affiliations:** Loschmidt Laboratories, Department of Experimental Biology and RECETOX, Faculty of Science, Masaryk University, 625 00 Brno, Czech Republic; Department of Information Systems, Faculty of Information Technology, Brno University of Technology, 612 00 Brno, Czech Republic; Loschmidt Laboratories, Department of Experimental Biology and RECETOX, Faculty of Science, Masaryk University, 625 00 Brno, Czech Republic; Department of Information Systems, Faculty of Information Technology, Brno University of Technology, 612 00 Brno, Czech Republic; International Clinical Research Centre, St. Anne’s University Hospital, 656 91 Brno, Czech Republic; Loschmidt Laboratories, Department of Experimental Biology and RECETOX, Faculty of Science, Masaryk University, 625 00 Brno, Czech Republic; International Clinical Research Centre, St. Anne’s University Hospital, 656 91 Brno, Czech Republic; Loschmidt Laboratories, Department of Experimental Biology and RECETOX, Faculty of Science, Masaryk University, 625 00 Brno, Czech Republic; International Clinical Research Centre, St. Anne’s University Hospital, 656 91 Brno, Czech Republic; Loschmidt Laboratories, Department of Experimental Biology and RECETOX, Faculty of Science, Masaryk University, 625 00 Brno, Czech Republic; International Clinical Research Centre, St. Anne’s University Hospital, 656 91 Brno, Czech Republic

## Abstract

**Summary:**

Protein design requires information about how mutations affect protein stability. Many web-based predictors are available for this purpose, yet comparing them or using them en masse is difficult. Here, we present BenchStab, a console tool/Python package for easy and quick execution of 19 predictors and result collection on a list of mutants. Moreover, the tool is easily extensible with additional predictors. We created an independent dataset derived from the FireProtDB and evaluated 24 different prediction methods.

**Availability and implementation:**

BenchStab is an open-source Python package available at https://github.com/loschmidt/BenchStab with a detailed README and example usage at https://loschmidt.chemi.muni.cz/benchstab. The BenchStab dataset is available on Zenodo: https://zenodo.org/records/10637728

## 1 Introduction

Protein stability is one of the key determinants of protein applicability. Stable proteins can withstand harsh industrial conditions such as high temperatures, unfavorable pH, or the presence of denaturing agents. However, most proteins have evolved to function in relatively mild environments (Modarres *et al.* 2016). Therefore, there is a need to engineer proteins to meet the requirements of commercial applications. The laborious and costly process of experimental methods can be partially mitigated using predictive tools that provide fast and inexpensive solutions for mutation prioritization. In recent years, the rise of machine-learning techniques and the availability of experimental data have led to a plethora of predictors of the effect of mutations on protein stability with varying accuracies, strengths, and weaknesses (Planas-Iglesias *et al.* 2021).

These predictors typically predict a change of Gibbs free energy (ΔΔG) or only classify mutations as stabilizing or destabilizing. Prediction may be based on structural information or sequence alone. We distinguish four basic modes of operations: (i) analysis of molecular interactions with force-field calculations (Yin *et al.* 2007), (ii) machine learning on structure-based features (Cheng *et al*. 2006), (iii) machine learning on features derived from a sequence (Folkman *et al.* 2016) or using a language model (Umerenkov *et al.* 2023), and (iv) meta predictions combining multiple other models (Chen *et al.* 2013). Particularly the number of predictors of the third type has risen recently thanks to breakthroughs in structure prediction and large language models for bioinformatic data (Umerenkov *et al.* 2023). We can expect a further increase in the number of predictors with the emergence of very large mutational datasets collected in a high-throughput manner (Tsuboyama *et al.* 2023).

For a selection of the best tools for protein engineering and establishing new predictive methods, proper and independent benchmarking is crucial. However, the large number of existing tools makes their comprehensive evaluation challenging. On the one hand, such evaluation can prove difficult due to the potential overlaps between training and test datasets, various formats of the input data, and provided outputs. On the other hand, a majority of machine learning predictors are only available as web services with limited input size, variable waiting times, and occasional downtimes, thus making a large-scale analysis a troublesome task.

Here, we present BenchStab, a freely available Python package for the swift execution of calculations on web-based predictors and collection of results. Our package currently implements 19 web-based computational tools that we evaluated on the independent dataset (Velecký *et al.* 2024) derived from FireProt^DB^ (Stourac *et al.* 2021).

BenchStab is fully modular, facilitating the integration of new web tools. We offer a straightforward solution for a fast and effective benchmarking of well-established and future tools for predicting the effect of mutations on protein stability. BenchStab represents a significant step toward a comprehensive evaluation of computational tools, identification of their limitations, and further advancement of the field of stability prediction using machine learning. We believe that our tool will be particularly useful to the machine learning community, as BenchStab may eliminate some barriers to entry into the competition of stability change prediction.

## 2 Implementation

**Figure 1.**
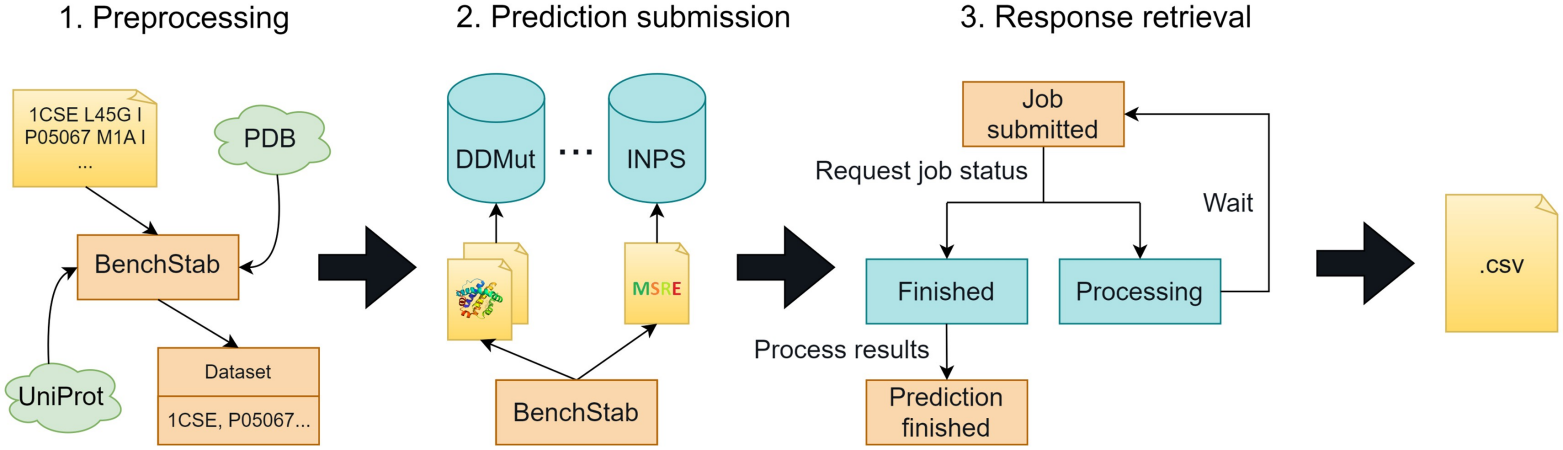
Three stages of the prediction acquisition process. The initial stage is the dataset preprocessing, validation, and enrichment. Every datapoint is then submitted to all selected predictors in the specific format unique to each tool. This is done asynchronously to minimize idling of the program as well as the user’s waiting since the responses can be handled immediately as they come (predictors without job queues) or awaited in a non-blocking loop (job-based predictors). Finally, the results are progressively merged as they are processed and periodically exported as a CSV file.

We developed BenchStab as a Python library with a command-line interface, fully automating the process of submitting requests to protein stability predictors and retrieving the results. The standalone application consists of multiple clients for distinct web-based predictors and allows adding new predictor clients through its framework, which comprises two main modules: (i) input data preprocessing and (ii) predictor client implementations (Fig. 1). The predictors upon a point mutation may be both classifiers and regression tools. The robustness of our application is proven by an automated test suite of 61 different unit tests. These tests also facilitate future application extensions with new predictors or other improvements.

Every BenchStab run involves preprocessing the input data using the pandas library (McKinney 2010). The input contains the list of mutations defined within a single file that adheres to a fixed column structure. The application accepts common column separators (commas, semicolons, tabs, spaces). Each row may define the target protein by a Protein Data Bank (PDB) or UniProt accession code, PDB file, FASTA file, or raw sequence. Users can also define specific temperature and pH values as per-row optional parameters so the values are forwarded to predictors that support them. Then, the tool performs cascade data acquisition to query each predictor with its required input, e.g. by retrieving a sequence for an entry specified by a PDB code for sequence-only tools. Where needed, SIFTS JSON API (Dana *et al.* 2019) is employed to map a PDB chain to UniProt and altogether with RCSB API (Rose *et al.* 2021), a correct mutation position in the sequence is calculated addressing PDB artifacts, such as insertion codes or expression tags. In the case of PDB files, the sequence is extracted directly from the file using Biopython. The integrity of submitted proteins, chains, and mutations is checked during preprocessing to ensure the predictors are not queried with faulty requests.

A client for a new predictor can be added using the adaptable framework implemented in our tool by following the steps described in the README file. The framework supports various protein data types, payload formats, authentication, and job-waiting loops. Moreover, it leverages both aiohttp and asyncio libraries, enabling a non-blocking communication between a client and the corresponding predictor and parallel processing of the input data, both predictor-wise and entry-wise. Additionally, our tool provides users with a collection of global and per-predictor options through a configuration file described in the documentation.

## 3 Results

We implemented the clients for 19 web-based tools out of 28 considered Supplementary Table S1. The remaining tools were not implemented due to (i) email-only results: STRUM (Quan *et al.* 2016), (ii) excessive job waiting times: ELASCPIC (Witvliet *et al.* 2016), (iii) malfunctioning prediction submission forms: EASE-MM (Folkman *et al.* 2016), (iv) server discontinuation: ENCoM, (Frappier *et al.* 2015), or (v) frequent outages and failures.

The sequence-based tools implemented in BenchStab are, with one exception, structure enabled. They offer two modes for prediction: from a sequence or a structure. In BenchStab, they are implemented as separate predictors, bringing the total number of available predictors to 25 (Supplementary Fig. S1). BenchStab can be set to query only the sequence-based or structure-enabled predictors.

We tested the proper function of the predictors and their integration within the tool as a potential use case on a crafted dataset. Prediction gathering consisted of several rounds of predictor queries during which we adjusted client parameters per predictor: the status-check delays, number of concurrent queries, and error handling (to avoid causing a denial of service).

## 4 Use case

BenchStab can be utilized to benchmark the available predictors on a specific mutational dataset. To demonstrate this functionality, we created a new dataset based on FireProt^DB^, disjoint from the commonly used datasets. We present the results collected using BenchStab on this dataset.

We used only the records with both ΔΔG measurements and PDB accession codes. To prevent data leakage from training datasets, we eliminated records similar to the proteins used in the training of the predictors as follows. First, we pooled all training datasets from the implemented predictors (Supplementary Table S2) to create a joint training set. Next, we assigned a UniRef50 cluster (Suzek *et al.* 2015) to each datapoint in both filtered FireProt^DB^ and training set. Finally, with assigned clusters, we eliminated all datapoints assigned any UniRef50 cluster ID appearing in the training set too. The resulting dataset comprises 289 records for 36 proteins (Velecký *et al.* 2024).

To check the structural heterogeneity of this dataset, we employed SCOP (Andreeva *et al.* 2014) for fold-based structure clustering to discover that our dataset contains 25 unique SCOP folds among the 36 proteins. Half of the folds were seen before by at least one of the predictors (Supplementary Table S3). Moreover, a distribution analysis shows that the dataset is not biased to a particular protein, an enzyme class, a particular structural element, or a conservation of mutated residues (Supplementary Fig. S2). However, the alanine-involving mutations make up half of the dataset, and many substitutions are not represented (Supplementary Fig. S3), which is a known problem for protein stability datasets (Caldararu *et al.* 2020). We explored a possible remedy by deriving new datapoints using thermodynamic permutation (Diaz *et al.* 2024), but only two structures for mutants in our dataset were available in the PDB at the time of writing. Further statistics on the produced dataset are presented in Supplementary Tables S3 and S4 and Supplementary Fig. S4.

With the dataset, we benchmarked 24 predictors: 22 of the predictors implemented in BenchStab (Supplementary Table S1) and two standalone tools — FoldX versions 4 and 5 (Schymkowitz *et al.* 2005) — providing a comparison with a popular standalone and force-field-based predictor. We did not include three of the implemented tools in the final results: sRide (Magyar *et al.* 2005), SDM (Worth *et al.* 2011), and PROSTATA (Umerenkov *et al.* 2023). The first does not provide predictions for individual mutants, the second became unavailable during benchmarking, and the last used heterogeneous training data including individual protein domains (Tsuboyama *et al.* 2023); creating a dataset robust to structural leakage via domains to guarantee a fair evaluation was beyond the scope of this study. Supplementary Figure S1 clarifies which tools were implemented and which were benchmarked.

**Figure 2.**
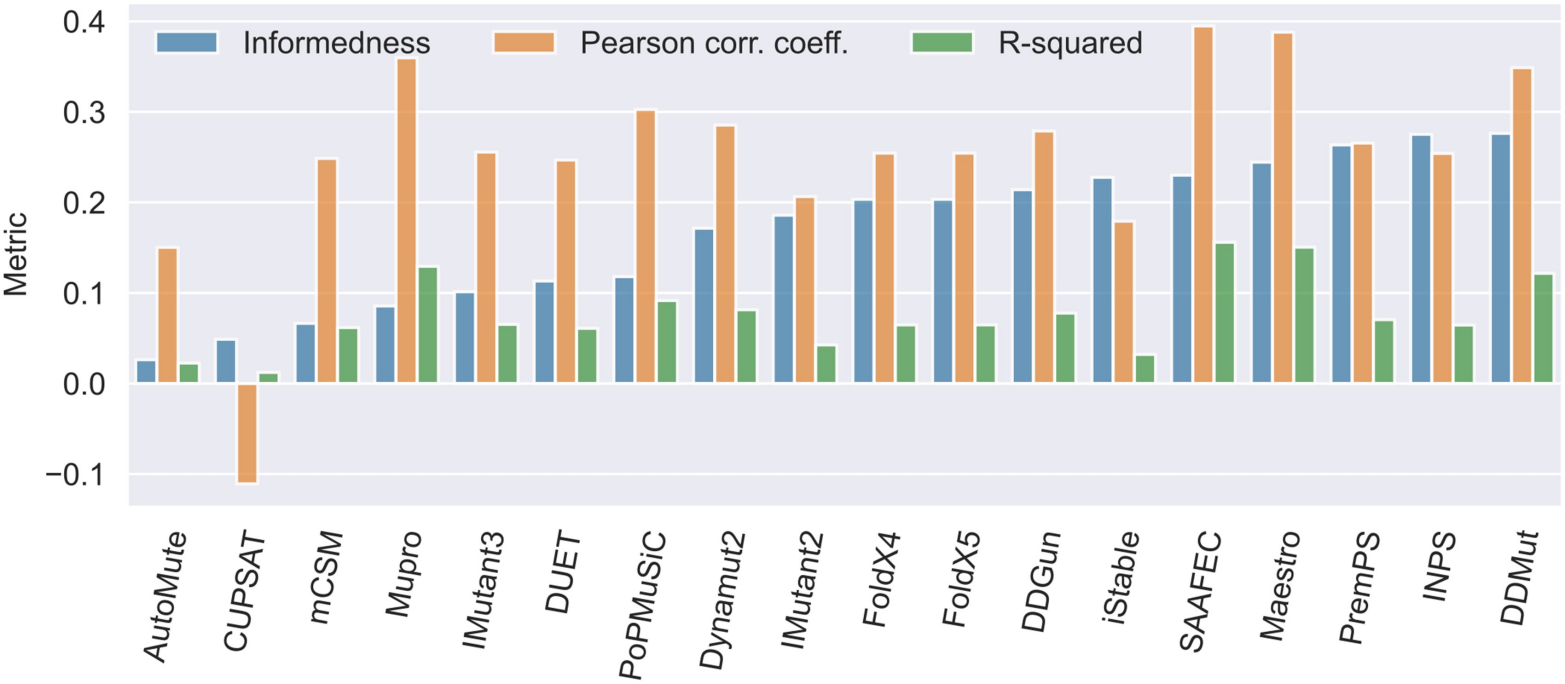
Performance of the predictors as measured on the BenchStab dataset. The tools are compared among themselves by these metrics: informedness, Pearson correlation coefficient, and R^2^. Informedness* (Powers 2011), a probability of an informed decision, is used to order the results. For the predictors with two input variants (structure and sequence), we selected the higher-scoring variant. *informedness [−1, 1] = 2 × balanced accuracy − 1 = recall + inverse recall − 1 = TP/P + TN/N − 1 where TP, TN stands for true positives, true negatives, and T, F for all true, false cases, respectively.

The concise statistics of the results are shown in Fig. 2 for both regression and binary classification (informedness; Powers 2011). Our evaluation revealed that most of the tools can be more or less successfully used for mutation prioritization with balanced accuracy between 51% and 64%. On the other hand, the overall low predictive performance (Supplementary Figs S5 and S6) implied considerable room for improvement. Almost all the tools showed a particularly poor performance in the regression task, i.e. predicting the exact change in the protein stability (the worst and best R^2^ equal to 0.01 and 0.15, respectively) with frequent both false positive and false negative errors (Supplementary Fig. S5). Furthermore, the vast majority of tested tools displayed a bias toward destabilizing predictions (Supplementary Fig. S8), also shown by mean signed deviation ranging from −0.79 to −0.11, as has been reported previously (Usmanova *et al.* 2018, Broom *et al.* 2020, Sanavia *et al.* 2020, Pucci *et al.* 2022). The abovementioned metrics, as well as root mean squared error, mean absolute error, accuracy, and Matthews or Pearson correlation coefficients, are reported for individual predictors in Supplementary Table S5. The structure-enabled tools did not perform much better than the sequence-only tools. In the case of precision-recall curves for binary classification, structure-enabled sequence-based predictors performed worse when the structures were provided (Supplementary Fig. S7), as was observed in another recent study (Pancotti *et al.* 2022).

## 5 Conclusions

We presented BenchStab – a tool that facilitates the use of online stability-change predictors and streamlines the process of benchmarking a new predictor against established competitors. Protein engineers can use it directly on their proteins of interest with a tailored dataset to find the best-working predictor in their use case. Our tool is validated by automated tests. On top of that, we investigated the robustness of our tool and of the underlying predictors on a newly created independent dataset.

As we can expect the discontinuation of some of the predictors in the future or breaking changes in their web interfaces, we released BenchStab as an open source to encourage quick updates from the scientific community. In the same way, our application could be extended to incorporate new predictors, including those for other protein properties, e.g. melting temperature or solubility.

We demonstrated the use case of the tool on a benchmarking task. The results revealed that hard cases for the current predictors exist, and therefore there is still a need for more precise tools. Structure-based tools did not beat their sequence-only counterparts. This finding seems consistent with a recent study (Pancotti *et al.* 2022) and may suggest that the structural information may not have been grasped optimally. We also reconfirmed the bias toward destabilizing predictions (Usmanova *et al.* 2018, Broom *et al.* 2020, Sanavia *et al.* 2020, Pucci *et al.* 2022). The dataset consists of proteins unseen by the benchmarked predictors before.

It is important to stress that the purpose of our dataset was to serve as test data and a use case for the BenchStab tool. Our dataset has several limitations, e.g. data from alanine-scanning experiments are overrepresented, which are often employed to identify residues crucially contributing to the protein stability (Caldararu *et al.* 2020), and several mutation types are not represented. Applying thermodynamic permutation (Diaz *et al.* 2024) to recover some mutation types would have a limited effect due to the unavailability of structures needed to query most of the predictors. Therefore, a more robust dataset is required for a comprehensive comparison of the predictors, which is beyond the scope of this study.

In conclusion, we believe BenchStab will motivate computer scientists to enter the domain of stability-change prediction by facilitating the comparison of their predictors to the state of the art.

## Supplementary Material

btae553_Supplementary_Data

## Data Availability

The data underlying this article are available in its online supplementary material. The BenchStab dataset is available on Zenodo: https://zenodo.org/records/10637728.
