# Supplementary material for: BenchStab: a tool for automated querying of web-based stability predictors
Source: Bioinformatics. 2024 Sep 11;40(9):btae553. doi: 10.1093/bioinformatics/btae553 (PMC11427696; doi:10.1093/bioinformatics/btae553)
Supplement: btae553_Supplementary_Data [file btae553_supplementary_data.pdf]

Supporting Information

BenchStab: a tool for automated querying of web-based stability predictors

Jan Velecký[1,#], Matej Berezný[2,#], Miloš Musil[1,2,3], Jiri Damborsky[1,3], David Bednar[1,3,*], Stanislav Mazurenko[1,3,*]

# Joint first authors, * Corresponding authors: 222755@mail.muni.cz, mazurenko@mail.muni.cz
[1] Loschmidt Laboratories, Department of Experimental Biology and RECETOX, Faculty of Science, Masaryk University, Kamenice 5, bld. C13, 625 00 Brno, Czech Republic
[2] Department of Information Systems, Faculty of Information Technology, Brno University of Technology, Czech Republic
[3] International Clinical Research Centre, St. Anne's University Hospital, Pekařská 53, Brno, Czech Republic

Table S1: The predictors considered in this study and their corresponding input options. The predictors can be divided by the number of mutations accepted in an input into two categories: single-mutation predictors (a query consists of one protein and one mutation) and mutant-list predictors (a query consists of one or more mutants for a single protein). We measured the prediction time in October 2023 for all successful (without timeouts or failures) predictions on our dataset. The time may have been affected by the predictors' queue sizes at the query time (including wait time). The color in the last column highlights fast (green, <2 min) and slow (orange, >10 min) predictors.

Predictor name	Implemented	Accepted mutation format	Accepted input	Prediction method**	Avg. prediction time per mutant
AutoMute [1]	✓	Single mutation	PDB ID	Several (SVM/RF/tree regression)	17 s
CUPsat [2]	✓		PDB ID	Linear regression	7 s
DDGun [3]	✓		PDB ID, PDB file, sequence	Linear regression	82 s
DUET [4]	✓		PDB ID, PDB file	Meta predictor (mCSM+SDM)	41 s*
I-Mutant2.0 [5]	✓		PDB ID, sequence	Support vector machine	80 s
I-Mutant3.0	✓		PDB ID, PDB file, sequence	Support vector machine	-
iStable [6]	✓		PDB ID, sequence	Meta predictor (5 base predictors)	2 s*
MUpro [7]	✓		PDB file, sequence	Support vector machine	5 s
PROSTATA [8]	✓		sequence	Deep learning: fine-tuned LLM (ESM-2)	30 s
pSTAB [9]	✗		PDB ID, PDB file	Physical force field	-
DynaMut2 [10]	✓	List of mutants	PDB ID, PDB file	Random forest	2.7 min
DDMut [11]	✓		PDB ID, PDB file	Deep learning: Siamese CNN	119 s
DeepDDG [12]	✗		PDB ID, PDB file	Deep learning: dense NN	-
EASE-MM [13]	✗		sequence	Ensemble of SVMs	-
ELASPIC [14]	✗		PDB ID, PDB file	Stochastic-gradient boosted DT	-
ENCoM [15]	✗		PDB ID, PDB file	Normal mode analysis	-
Eris [16]	✗		PDB ID, PDB file	Physical force field (Medusa)	-
INPS [17]	✓		PDB ID, sequence	Support vector machine	4.5 min
Maestro [18]	✓		PDB ID	Consensus (NN, SVM, linear regression)	20 s
mCSM [19]	✓		PDB file	Gaussian process regression	23 s
PON-tstab [20]	✗		PDB file	Random forest	-
PoPMuSiC [21]	✓		PDB ID	Neural network	6 min
PremPS [22]	✓		PDB ID, PDB file	Random forest	8.3 min
SDM 2.0 [23]	✓		PDB ID, PDB file	Environment-specific substitution tables	-
SAAFEC-SEQ [24]	✓		sequence	Gradient boosted decision tree	39 min
STRUM [25]	✗		PDB file, sequence	Gradient boosted decision tree	-
TKSA-MC [26]	✗		PDB ID, PDB file	Monte Carlo, Tanford–Kirkwood method	-
sRIDE [27]	✓	N/A	PDB ID, PDB file	Based on cut-offs of structural features	92 s

**The predictor utilizes caching, i.e., provides faster results for repeated inputs. **CNN = Convolutional NN, DT = decision tree, LLM = large language model, NN = neural network, RF = random forest, SVM = support vector machine*

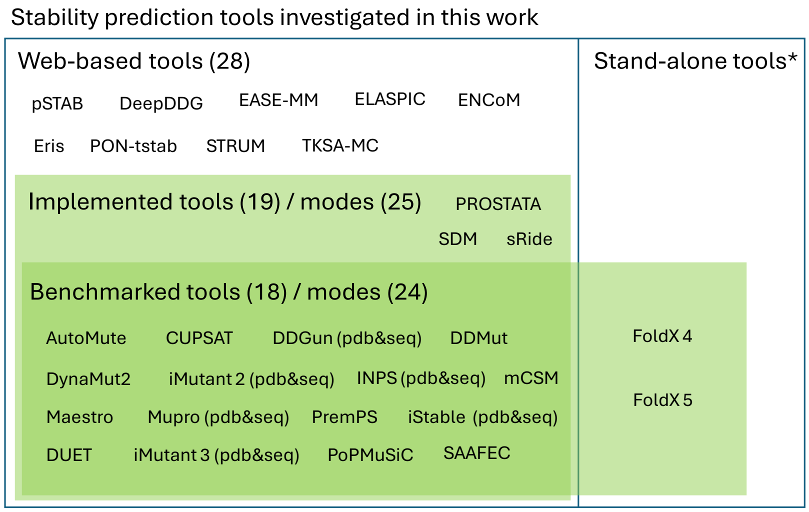

*Figure S1: Venn diagram of prediction tools investigated in this work. Some of the web tools were not implemented in BenchStab, e.g. due to their unavailability. The investigated/implemented web predictors are listed in greater detail in Table S1 and the benchmarked predictors in Table S2. The numbers for the respective groups are counts of the predictor tools / predictor modes. In the latter, we count sequence and structural modes separately. *Stand-alone prediction tools were not the focus of this work.*

Dataset statistics

Table S2: List of training (machine learning), or training and validation (force-fields) datasets used during the development of the benchmarked predictors. They were all excluded from our benchmark dataset based on sequence similarity (using UniRef50 clusters of UniProt release 2023-01).

Predictor/Dataset	S2648	S1603	P53	S350	S1765	S1538	S1948	S1925	S1615	S2298	S4022	S9028
CUPsat [2]	✗	✓	✗	✗	✗	✓	✗	✗	✗	✗	✗	✗
DUET [4]	✓	✗	✗	✗	✗	✗	✗	✗	✗	✗	✗	✗
I-Mutant2.0 [5]	✗	✗	✗	✗	✗	✗	✓	✗	✗	✗	✗	✗
iStable [6]	✓	✗	✗	✗	✗	✗	✓	✗	✗	✗	✗	✗
MUpro [7]	✗	✗	✗	✗	✗	✗	✗	✗	✓	✗	✗	✗
DynaMut2 [9]	✓	✗	✗	✓	✗	✗	✗	✗	✗	✓	✓	✗
DDMut [10]	✗	✗	✗	✗	✗	✗	✗	✗	✗	✗	✗	✓
INPS [16]	✓	✗	✓	✗	✗	✗	✗	✗	✗	✗	✗	✗
Maestro [17]	✓	✗	✗	✓	✓	✗	✗	✓	✗	✗	✗	✗
mCSM [18]	✓	✗	✗	✓	✗	✗	✗	✓	✗	✗	✗	✗
PoPMuSiC [20]	✓	✗	✗	✗	✗	✗	✗	✗	✗	✗	✗	✗
PremPS [21]	✓	✗	✗	✗	✗	✗	✗	✗	✗	✗	✗	✗
SDM 2.0 [22]	✓	✗	✗	✓	✗	✗	✗	✗	✗	✗	✗	✗
SAAFEC-SEQ [24]	✓	✗	✓	✓	✗	✗	✗	✗	✗	✗	✗	✗
AutoMute [1]	N/A, extracted from FireProtDB											
I-Mutant3.0												
DDGun [3]	Untrained											

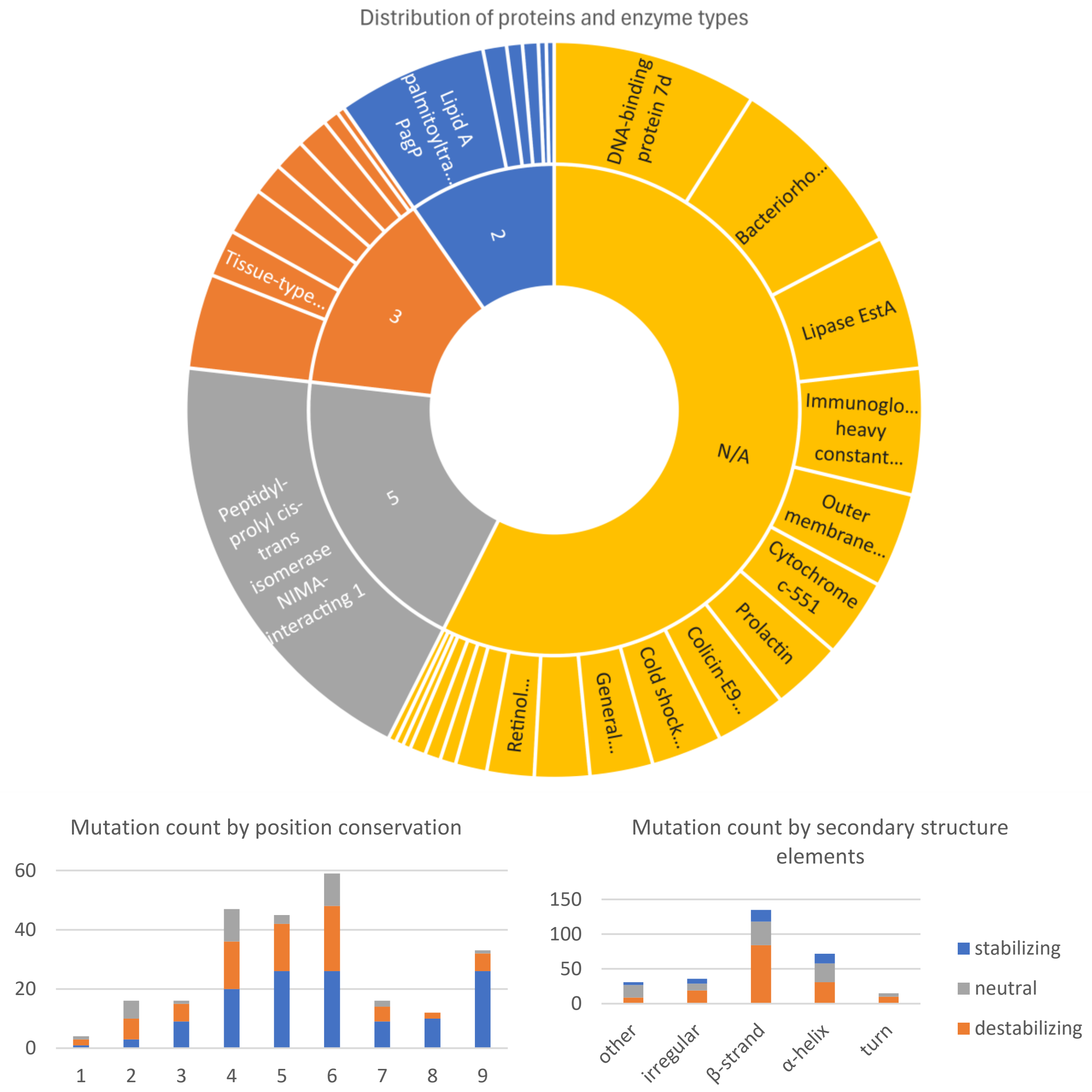

Figure S2: Distributions of proteins, conservation, structural elements by datapoints. The top chart shows distributions in our dataset: first-level Enzyme Commission numbers (inside) and individual proteins (outside). More than half of the included proteins (N/A) do not have an enzyme number assigned, and only 3 of 7 enzyme classes are present in the dataset. The conservation and secondary structure elements data are provided by HotSpot Wizard [28]. Conservation scores of 1–3, 4–5, and 6–9 indicate low, moderate, and high mutability, respectively. Mutations within the range -0.5–0.5 kcal/mol of ΔΔG are labeled neutral.

Table S3: Basic summary of the BenchStab dataset used to compare the predictors. We created the dataset through a series of filtering steps from FireProtDB [29]. More than half of those SCOP families in our dataset are not present in the excluded training sets.

Mutations	289
Stabilizing mutations	80 (28 %)
Represented amino-acid substitutions	89/380 (23 %)
Proteins	36
Uniref50 clusters	35
SCOP folds	25
Unique to the dataset	13 (52 %)
SCOP families	31
Unique to the dataset	18 (58 %)

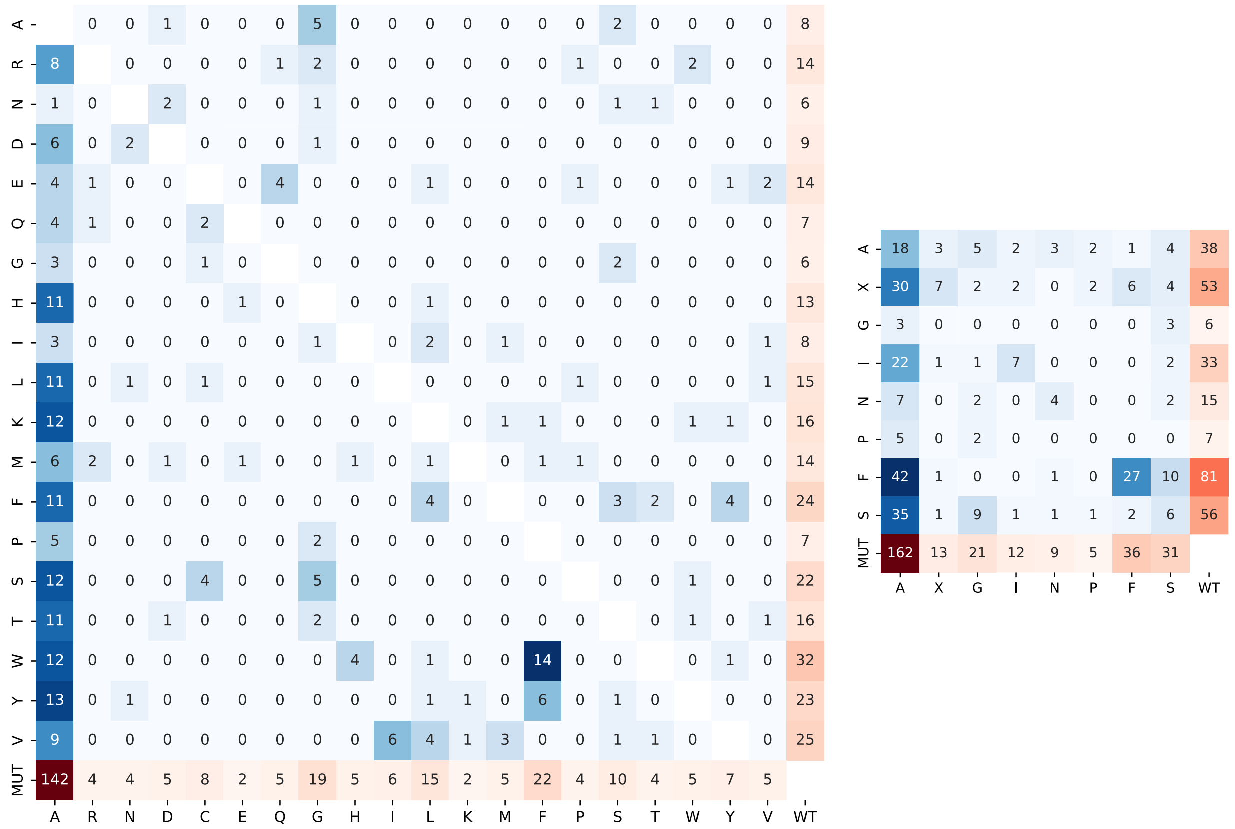

Figure S3: Amino-acid substitution matrices. The left matrix displays counts of amino-acid substitutions from (rows) to (columns) represented in the dataset. The right matrix displays counts of substitutions between amino-acid groups in a reduced alphabet as per [30]. The column and the row tinged in red are sums of substitutions from and to particular amino acids respectively. Mutations to alanine represent half of the dataset.

Table S4: Summary of the benchmark dataset based on the properties of target amino acids, according to the international ImMunoGeneTics information system® [30].

Amino-acid property	Property classes	Count
Charge	Positive	11
	Negative	7
	Neutral	271
Chemical	Acidic	7
	Amide	9
	Aliphatic	195
	Basic	11
	Sulfur	15
	Hydroxyl	16
Polarity	Polar	41
	Non-polar	248

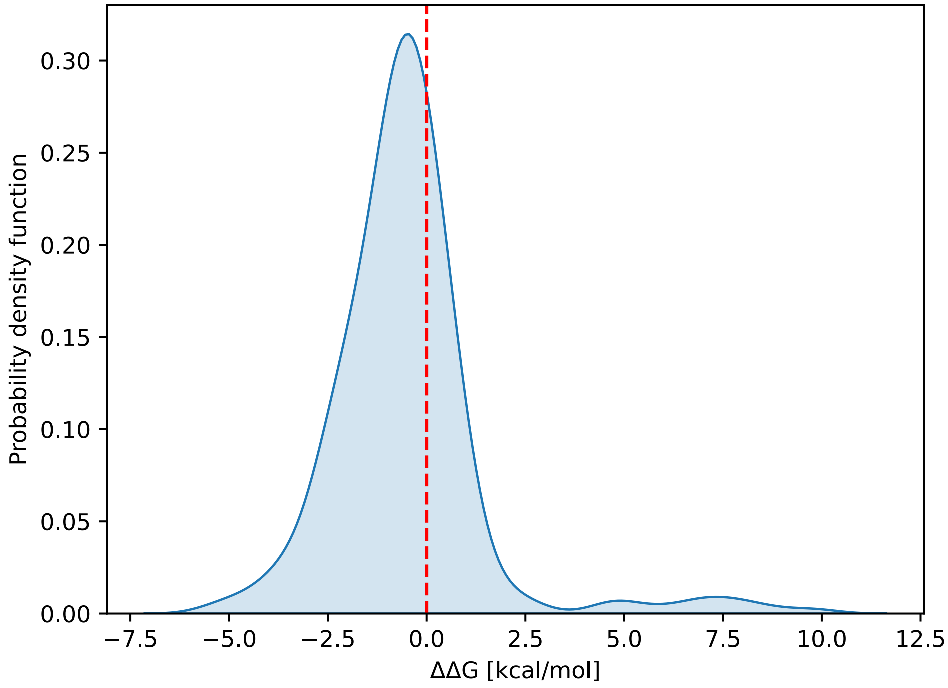

Figure S4: Kernel density estimate plot of experimentally determined ΔΔG distribution of the benchmark dataset (ground truth). Negative ΔΔG corresponds to destabilizing mutations.

BenchStab use case

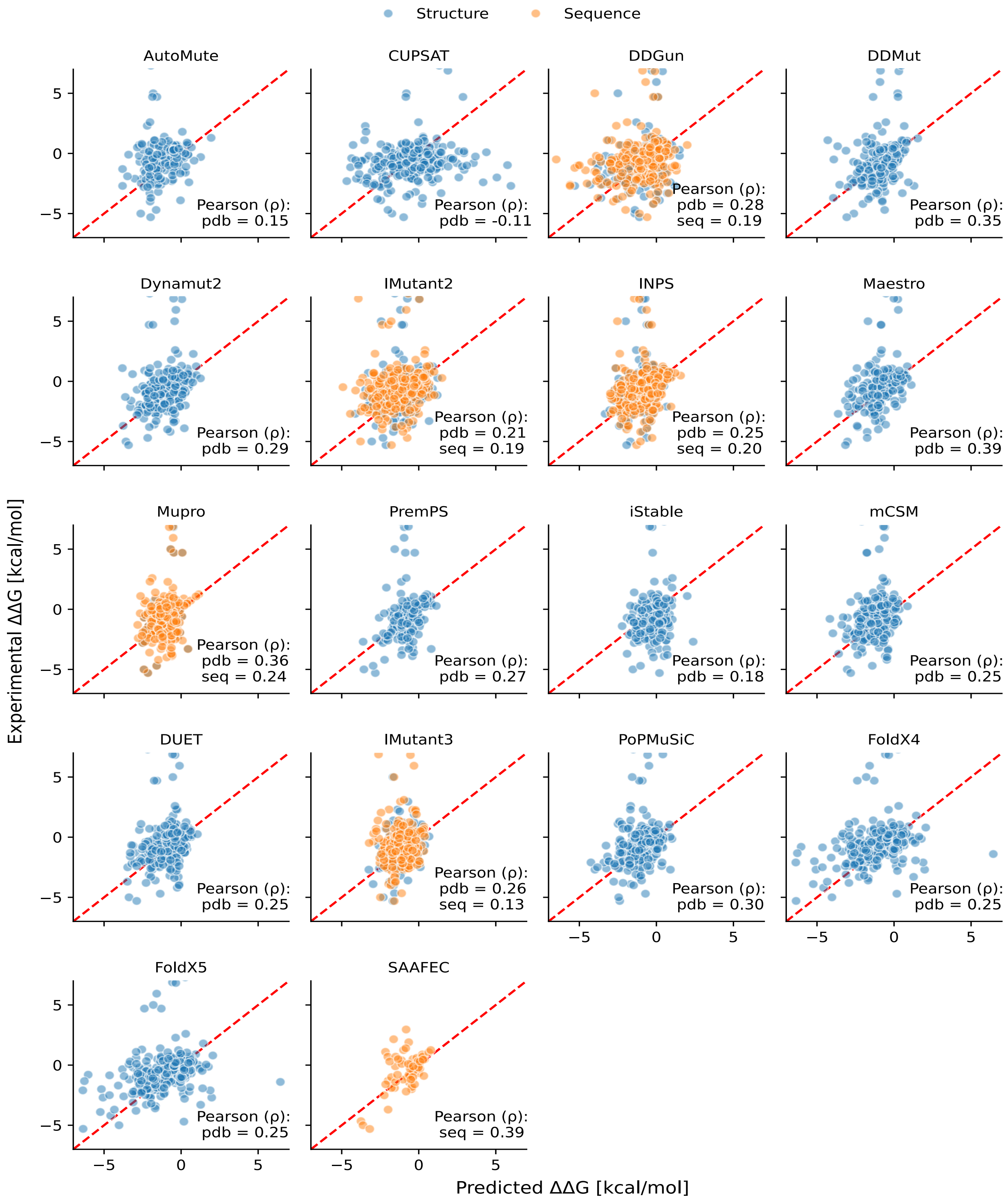

Figure S5: Correlation graphs between predicted ΔΔG (kcal/mol) and the ground truth. Each subgraph corresponds to one tool. Each dot corresponds to a prediction for a particular mutant either from a protein sequence (orange, seq) or a protein structure (blue, pdb). The red dashed line represents the ideal prediction. For the presentation purpose, the subgraphs are clipped to range [-7, 7], but the outlying points are still included in the calculation of the Pearson correlation coefficients. We can observe, from the positions of the central clusters which are shifted above the diagonals, that many predictors predict more negative effects than is the ground truth, e.g. AutoMute, mCSM. This suggests a bias toward the prediction of destabilizing mutations (negative ΔΔG). Also notably, the predictors mispredict a cluster of phenylalanine and cysteine mutants (middle-top in the graphs), which have very high stabilizing experimental ΔΔG (~5 kcal/mol), as neutral (ΔΔG close to 0).

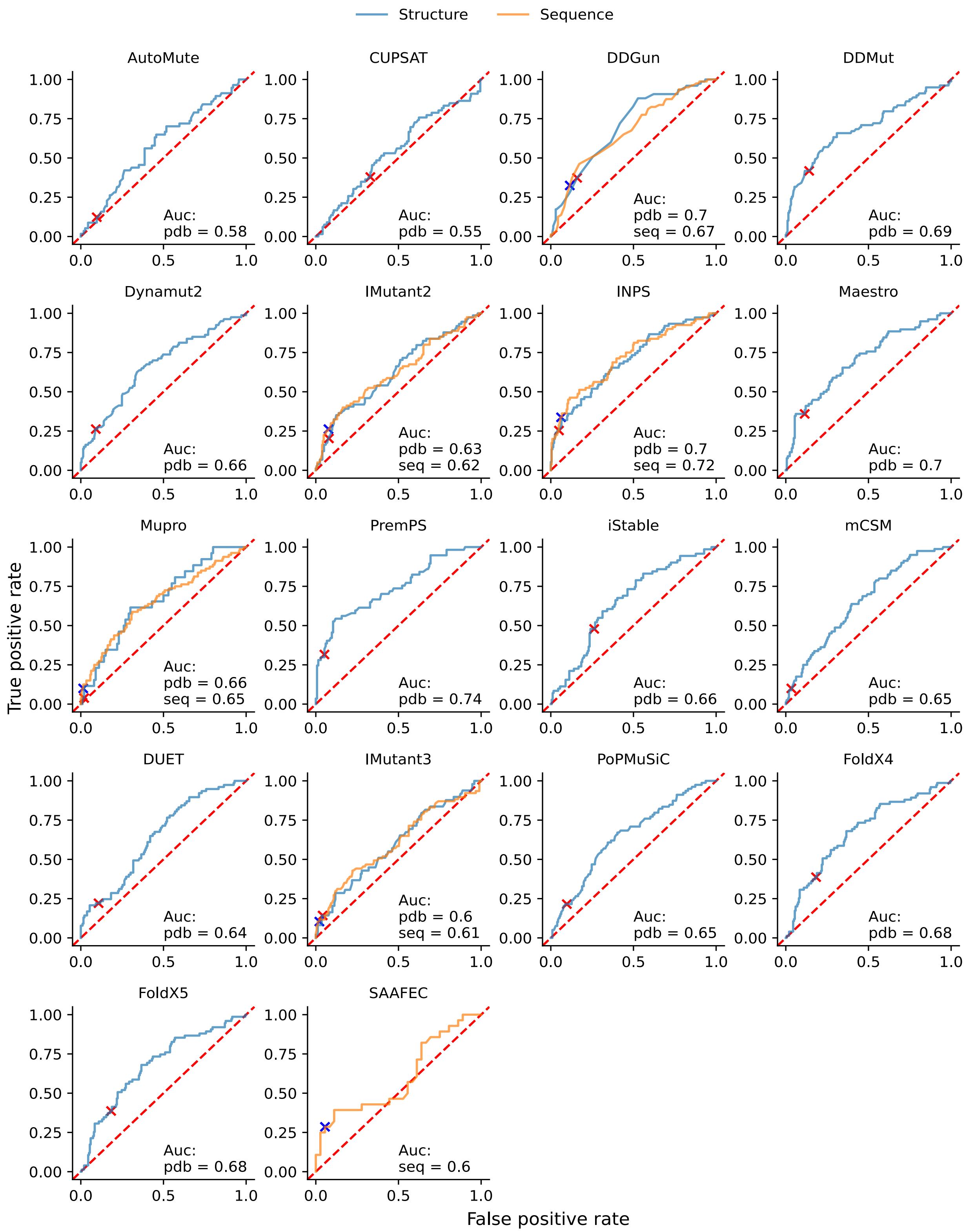

Figure S6: ROC curves depicting the relationship between false positive rate and true positive rate (sensitivity/recall) at different thresholds to classify mutations as stabilizing or destabilizing. The blue curve represents the structural mode of the predictor, the orange curve is its sequence-based mode, and the red dashed line is the random predictor. The default threshold (0 kcal/mol) is marked with a red or blue cross for structural or sequence-based modes respectively.

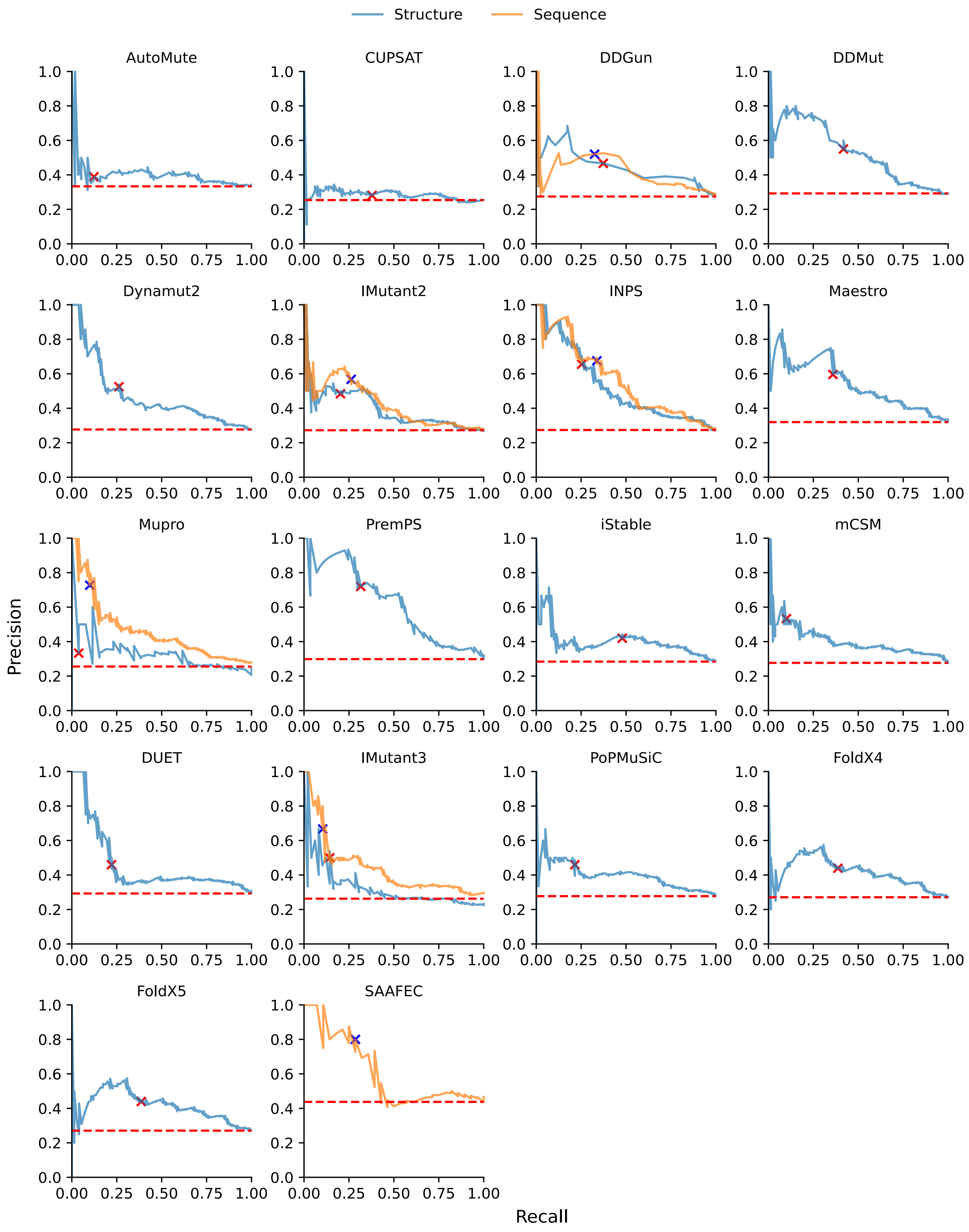

Figure S7: Precision-recall curves depict the relationship between precision and recall at different thresholds to classify mutations as stabilizing or destabilizing. The blue curve represents the structural mode of the predictor, the orange curve is its sequence-based mode, and the red dashed line is the random predictor adjusted to the pool of data successfully processed by the given tool. The default threshold (0 kcal/mol) is marked with a red or blue cross for structural or sequence-based modes respectively.

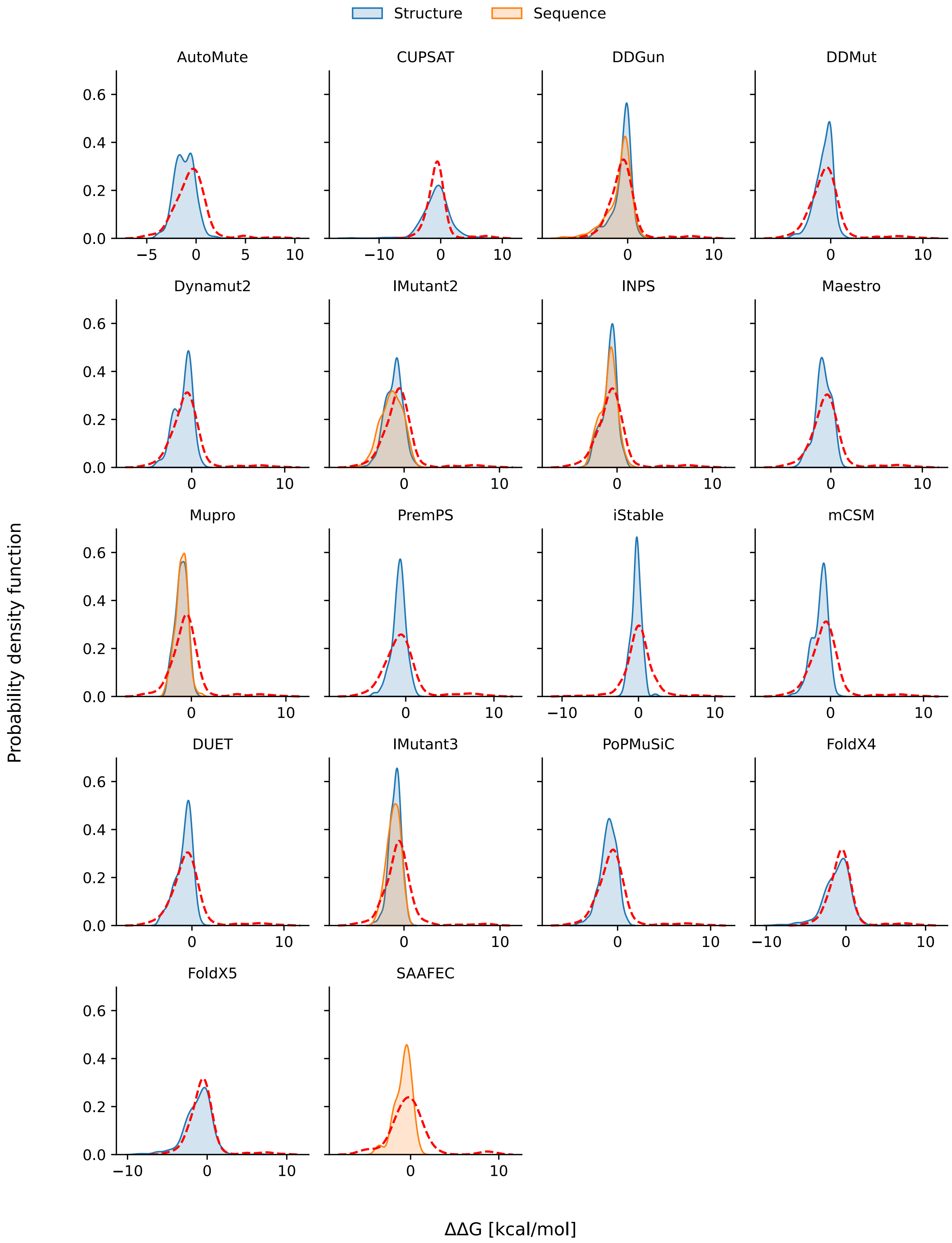

Figure S8: Kernel density estimate plot depicting the predicted ΔΔG distributions per predictor and input type. Orange represents predictors predicting from a sequence, while blue represents tools predicting from a structure. The red dashed line shows the ΔΔG distribution of a subset of ground truth labels belonging to successfully predicted data points. Most of the tools displayed a substantial shift of their distributions leftwards to their respective ground truth distribution, i.e. pointing on a bias towards destabilizing predictions (negative ΔΔG values).

*Table S5: Assessment of the web-based protein stability prediction tools on our benchmark dataset derived from FireProtDB. The following metrics were used: root mean squared error (**RMSE**), mean absolute error (**MAE**), mean signed deviation (**MSD**), highlighting a potential bias toward destabilizing/stabilizing mutations, binary accuracy and balanced accuracy (**BA**), Pearson correlation coefficient (**PCC**), Matthews correlation coefficient (**MCC**), and R-squared (**R^2**). We used the cut-off of 0 kcal/mol for the binary classification. Results were collected in October 2023 if not stated otherwise. For the calculation of FoldX results, we used the 2024 Mac binaries with the RepairPDB module, followed by the PssmStability module setting pH equal to 7 and --water=CRYSTAL. The green or orange color represents a good or bad (see below for the cut-offs) performer respectively for a given metric. The best performers are marked by their place in parentheses.*

Predictor name	Input type	Method	# of results	RMSE	MAE	MSD***	Accuracy	BA	PCC	MCC	R^2
AutoMute	PDB ID	Machine Learning	171	1.98	1.31	-0.63	0.64	0.51	0.15	0.04	0.02
CUPSAT	PDB ID	Free Energy	260	3.14	1.95	-0.22	0.6	0.52	-0.11	0.04	0.01
DDGun	PDB ID	Free Energy	276	1.93	1.19	-0.11	0.71	0.61	0.28	0.23	0.08
	sequence**		289	2.23	1.44	-0.45	0.73	0.6	0.19	0.25	0.04
DDMut	PDB ID	Machine Learning	270	1.89	1.11	-0.25	0.73	0.64 (1-2)	0.35	0.3	0.12
DUET	PDB ID	Meta approach	263	2.02	1.22	-0.41	0.7	0.56	0.25	0.15	0.06
Dynamut2	PDB ID	Meta approach	289	1.93	1.14	-0.4	0.73	0.59	0.29	0.22	0.08
FoldX4[31]	PDB ID	Free Energy	277	2.23	1.40	-0.63	0.7	0.6	0.25	0.21	0.06
FoldX5[31]	PDB ID	Free Energy	277	2.23	1.40	-0.63	0.7	0.6	0.25	0.21	0.06
IMutant2	PDB ID	Machine Learning	276	2.04	1.23	-0.58	0.73	0.56	0.21	0.17	0.04
	sequence**		289	2.2	1.42	-0.79	0.74	0.59	0.19	0.25	0.04
IMutant3*	PDB ID	Machine Learning	218	1.53	0.94	-0.25	0.78 (2)	0.55	0.26	0.17	0.06
	sequence		262	2.11	1.35	-0.68	0.72	0.54	0.13	0.18	0.02
INPS	PDB ID	Machine Learning	277	1.92	1.08	-0.31	0.76	0.6	0.25	0.3	0.06
	sequence		289	1.98	1.17	-0.32	0.77 (3)	0.64 (1-2)	0.2	0.35 (3)	0.04
Maestro	PDB ID	Meta approach	244	1.92	1.15	-0.47	0.72	0.62	0.39 (1-2)	0.29	0.15 (1-2)
Mupro	PDB ID	Machine Learning	126	1.81	1.09	-0.57	0.79 (1)	0.51	0.36 (3)	0.05	0.13 (3)
	sequence**		289	1.94	1.25	-0.52	0.74	0.55	0.24	0.2	0.05
PoPMuSiC	PDB ID	Free Energy	285	1.92	1.16	-0.49	0.71	0.56	0.30	0.16	0.09
PremPS	PDB ID	Machine Learning	191	2.21	1.21	-0.33	0.76	0.63 (3)	0.27	0.36 (2)	0.07
SAAFEC*	sequence	Machine Learning	64	2.12	1.23	-0.62	0.65	0.62	0.39 (1-2)	0.39 (1)	0.15 (1-2)
iStable	PDB ID	Meta approach	250	1.91	1.16	0.23	0.66	0.61	0.18	0.21	0.03
	sequence**		283	N/A	N/A	N/A	0.71	0.62	N/A	0.25	N/A
mCSM	PDB file	Machine Learning	289	2.0	1.22	-0.62	0.73	0.53	0.25	0.13	0.02

**Reported as collected in the previous run (April 2023), due to the unavailability of the predictor in October 2023. **Collected in February 2024. ***MSD = $\frac{1}{n}\sum_i \hat{y}_i - y_i$*

Cut-offs of good and bad performers: 1.8 < RMSE < 2.0, 1.2 < MAE < 1.5, 0.25 < |MSD| < 0.6 , 0.76 > Accuracy > 0.7, BA > 0.6, 0.3 > PCC/MCC > 0.2, R^2 > 0.

References

[1] M. Masso and I. I. Vaisman, “AUTO-MUTE: web-based tools for predicting stability changes in proteins due to single amino acid replacements,” *Protein Engineering, Design and Selection*, vol. 23, no. 8, pp. 683–687, Aug. 2010, doi: 10.1093/protein/gzq042.

[2] V. Parthiban, M. M. Gromiha, and D. Schomburg, “CUPSAT: prediction of protein stability upon point mutations,” *Nucleic Acids Research*, vol. 34, no. suppl_2, pp. W239–W242, Jul. 2006, doi: 10.1093/nar/gkl190.

[3] L. Montanucci *et al.*, “DDGun: an untrained predictor of protein stability changes upon amino acid variants,” *Nucleic Acids Research*, vol. 50, no. W1, pp. W222–W227, Jul. 2022, doi: 10.1093/nar/gkac325.

[4] D. E. V. Pires, D. B. Ascher, and T. L. Blundell, “DUET: a server for predicting effects of mutations on protein stability using an integrated computational approach,” *Nucleic Acids Research*, vol. 42, no. W1, pp. W314–W319, Jul. 2014, doi: 10.1093/nar/gku411.

[5] E. Capriotti, P. Fariselli, and R. Casadio, “I-Mutant2.0: predicting stability changes upon mutation from the protein sequence or structure,” *Nucleic Acids Research*, vol. 33, no. suppl_2, pp. W306–W310, Jul. 2005, doi: 10.1093/nar/gki375.

[6] C.-W. Chen, J. Lin, and Y.-W. Chu, “iStable: off-the-shelf predictor integration for predicting protein stability changes,” *BMC Bioinformatics*, vol. 14, no. 2, p. S5, Jan. 2013, doi: 10.1186/1471-2105-14-S2-S5.

[7] J. Cheng, A. Randall, and P. Baldi, “Prediction of protein stability changes for single-site mutations using support vector machines,” *Proteins: Structure, Function, and Bioinformatics*, vol. 62, no. 4, pp. 1125–1132, 2006, doi: 10.1002/prot.20810.

[8] D. Umerenkov *et al.*, “PROSTATA: a framework for protein stability assessment using transformers,” *Bioinformatics*, vol. 39, no. 11, p. btad671, Nov. 2023, doi: 10.1093/bioinformatics/btad671.

[9] S. Gopi, D. Devanshu, P. Krishna, and A. N. Naganathan, “pStab: prediction of stable mutants, unfolding curves, stability maps and protein electrostatic frustration,” *Bioinformatics*, vol. 34, no. 5, pp. 875–877, Mar. 2018, doi: 10.1093/bioinformatics/btx697.

[10] C. H. M. Rodrigues, D. E. V. Pires, and D. B. Ascher, “DynaMut2: Assessing changes in stability and flexibility upon single and multiple point missense mutations,” *Protein Science*, vol. 30, no. 1, pp. 60–69, 2021, doi: 10.1002/pro.3942.

[11] Y. Zhou, Q. Pan, D. E. V. Pires, C. H. M. Rodrigues, and D. B. Ascher, “DDMut: predicting effects of mutations on protein stability using deep learning,” *Nucleic Acids Research*, vol. 51, no. W1, pp. W122–W128, Jul. 2023, doi: 10.1093/nar/gkad472.

[12] H. Cao, J. Wang, L. He, Y. Qi, and J. Z. Zhang, “DeepDDG: Predicting the Stability Change of Protein Point Mutations Using Neural Networks,” *J. Chem. Inf. Model.*, vol. 59, no. 4, pp. 1508–1514, Apr. 2019, doi: 10.1021/acs.jcim.8b00697.

[13] L. Folkman, B. Stantic, A. Sattar, and Y. Zhou, “EASE-MM: Sequence-Based Prediction of Mutation-Induced Stability Changes with Feature-Based Multiple Models,” *Journal of Molecular Biology*, vol. 428, no. 6, pp. 1394–1405, Mar. 2016, doi: 10.1016/j.jmb.2016.01.012.

[14] D. K. Witvliet, A. Strokach, A. F. Giraldo-Forero, J. Teyra, R. Colak, and P. M. Kim, “ELASPIC web-server: proteome-wide structure-based prediction of mutation effects on protein stability and binding affinity,” *Bioinformatics*, vol. 32, no. 10, pp. 1589–1591, May 2016, doi: 10.1093/bioinformatics/btw031.

[15] V. Frappier, M. Chartier, and R. J. Najmanovich, “ENCoM server: exploring protein conformational space and the effect of mutations on protein function and stability,” *Nucleic Acids Research*, vol. 43, no. W1, pp. W395–W400, Jul. 2015, doi: 10.1093/nar/gkv343.

[16] S. Yin, F. Ding, and N. V. Dokholyan, “Eris: an automated estimator of protein stability,” *Nat Methods*, vol. 4, no. 6, pp. 466–467, Jun. 2007, doi: 10.1038/nmeth0607-466.

[17] C. Savojardo, P. Fariselli, P. L. Martelli, and R. Casadio, “INPS-MD: a web server to predict stability of protein variants from sequence and structure,” *Bioinformatics*, vol. 32, no. 16, pp. 2542–2544, Aug. 2016, doi: 10.1093/bioinformatics/btw192.

[18] J. Laimer, H. Hofer, M. Fritz, S. Wegenkittl, and P. Lackner, “MAESTRO - multi agent stability prediction upon point mutations,” *BMC Bioinformatics*, vol. 16, no. 1, p. 116, Apr. 2015, doi: 10.1186/s12859-015-0548-6.

[19] D. E. V. Pires, D. B. Ascher, and T. L. Blundell, “mCSM: predicting the effects of mutations in proteins using graph-based signatures,” *Bioinformatics*, vol. 30, no. 3, pp. 335–342, Feb. 2014, doi: 10.1093/bioinformatics/btt691.
[20] Y. Yang, S. Urolagin, A. Niroula, X. Ding, B. Shen, and M. Vihinen, “PON-tstab: Protein Variant Stability Predictor. Importance of Training Data Quality,” *International Journal of Molecular Sciences*, vol. 19, no. 4, Art. no. 4, Apr. 2018, doi: 10.3390/ijms19041009.
[21] Y. Dehouck, J. M. Kwasigroch, D. Gilis, and M. Rooman, “PoPMuSiC 2.1: a web server for the estimation of protein stability changes upon mutation and sequence optimality,” *BMC Bioinformatics*, vol. 12, no. 1, p. 151, May 2011, doi: 10.1186/1471-2105-12-151.
[22] Y. Chen, H. Lu, N. Zhang, Z. Zhu, S. Wang, and M. Li, “PremPS: Predicting the impact of missense mutations on protein stability,” *PLOS Computational Biology*, vol. 16, no. 12, p. e1008543, 12 2020, doi: 10.1371/journal.pcbi.1008543.
[23] A. P. Pandurangan, B. Ochoa-Montaño, D. B. Ascher, and T. L. Blundell, “SDM: a server for predicting effects of mutations on protein stability,” *Nucleic Acids Research*, vol. 45, no. W1, pp. W229–W235, Jul. 2017, doi: 10.1093/nar/gkx439.
[24] G. Li, S. K. Panday, and E. Alexov, “SAAFEC-SEQ: A Sequence-Based Method for Predicting the Effect of Single Point Mutations on Protein Thermodynamic Stability,” *International Journal of Molecular Sciences*, vol. 22, no. 2, Art. no. 2, Jan. 2021, doi: 10.3390/ijms22020606.
[25] L. Quan, Q. Lv, and Y. Zhang, “STRUM: structure-based prediction of protein stability changes upon single-point mutation,” *Bioinformatics*, vol. 32, no. 19, pp. 2936–2946, Oct. 2016, doi: 10.1093/bioinformatics/btw361.
[26] V. G. Contessoto, V. M. de Oliveira, B. R. Fernandes, G. G. Slade, and V. B. P. Leite, “TKSA-MC: A web server for rational mutation through the optimization of protein charge interactions,” *Proteins: Structure, Function, and Bioinformatics*, vol. 86, no. 11, pp. 1184–1188, 2018, doi: 10.1002/prot.25599.
[27] C. Magyar, M. M. Gromiha, G. Pujadas, G. E. Tusnády, and I. Simon, “SRide: a server for identifying stabilizing residues in proteins,” *Nucleic Acids Research*, vol. 33, no. suppl_2, pp. W303–W305, Jul. 2005, doi: 10.1093/nar/gki409.
[28] L. Sumbalova, J. Stourac, T. Martinek, D. Bednar, and J. Damborsky, “HotSpot Wizard 3.0: web server for automated design of mutations and smart libraries based on sequence input information,” *Nucleic Acids Research*, vol. 46, no. W1, pp. W356–W362, Jul. 2018, doi: 10.1093/nar/gky417.
[29] J. Stourac *et al.*, “FireProtDB: database of manually curated protein stability data,” *Nucleic Acids Research*, vol. 49, no. D1, pp. D319–D324, Jan. 2021, doi: 10.1093/nar/gkaa981.
[30] C. Pommié, S. Levadoux, R. Sabatier, G. Lefranc, and M.-P. Lefranc, “IMGT standardized criteria for statistical analysis of immunoglobulin V-REGION amino acid properties,” *Journal of Molecular Recognition*, vol. 17, no. 1, pp. 17–32, 2004, doi: 10.1002/jmr.647.
[31] J. Schymkowitz, J. Borg, F. Stricher, R. Nys, F. Rousseau, and L. Serrano, “The FoldX web server: an online force field,” *Nucleic Acids Research*, vol. 33, no. suppl_2, pp. W382–W388, Jul. 2005, doi: 10.1093/nar/gki387.
[32] O. Caldararu, R. Mehra, T. L. Blundell, and K. P. Kepp, “Systematic Investigation of the Data Set Dependency of Protein Stability Predictors,” *J. Chem. Inf. Model.*, vol. 60, no. 10, pp. 4772–4784, Oct. 2020, doi: 10.1021/acs.jcim.0c00591.
